# Tailoring advanced breast cancer treatment after cyclin-dependent kinase 4/6 inhibitors progression - real-world data analysis

**DOI:** 10.3389/fonc.2024.1408664

**Published:** 2024-06-07

**Authors:** Marcin Kubeczko, Anna Polakiewicz-Gilowska, Katarzyna Świderska, Aleksandra Leśniak, Marta Mianowska-Malec, Barbarba Łanoszka, Konstanty Chomik, Barbara Grandys, Natalya Lisovska, Barbara Bobek-Billewicz, Ewa Chmielik, Michał Jarząb

**Affiliations:** ^1^ Breast Cancer Center, Maria Sklodowska-Curie National Research Institute of Oncology, Gliwice, Poland; ^2^ Radiology and Diagnostic Imaging Department, Maria Sklodowska-Curie National Research Institute of Oncology, Gliwice, Poland; ^3^ Tumor Pathology Department, Maria Sklodowska-Curie National Research Institute of Oncology, Gliwice, Poland

**Keywords:** advanced breast cancer, cyclin-dependent kinase 4/6 inhibitors, disease progression, tailored treatment, ribociclib, palbociclib, abemaciclib, targeted therapy

## Abstract

**Background:**

Cyclin-dependent kinase 4/6 inhibitors (CDK4/6i) represent the gold standard of the hormone receptor positive human epidermal growth factor receptor 2 (HER-2) negative advanced breast cancer. However, optimal treatment after disease progression is a matter of debate. We aimed to assess predictive and prognostic factors associated with the treatment outcome following CDK4/6i progression.

**Methods:**

We retrospectively analyzed patients who progressed on CDK4/6i treatment between 2018 and 2024. Treatment based on molecular findings (PIK3CA mutation), genetic findings (BRCA1/2 germline mutation), or adapted to the change in the tumor phenotype in rebiopsy (anti-HER2 therapy in the transformation to HER-2-positive disease) was grouped into tailored treatment and compared to the endocrine-based therapy and chemotherapy alone.

**Results:**

Five hundred twelve patients were treated with CDK4/6i. Two hundred patients with disease progression were enrolled in the study. Duration of response to CDK4/6i was not predictive of the response to subsequent treatment, whereas the progression in the central nervous system was the worst prognostic factor. Thirty patients were ineligible for subsequent treatment. Survival after CDK4/6i progression was significantly longer in patients eligible for tailored treatment. The median PFS in patients with tailored treatment (n=19) was 13.5 months vs. 4.9 months in patients with non-tailored therapy (n=151; p=0.045). 12-month PFS was 54.1% with tailored treatment [95% CI 24.1–76.7%] compared to 18.5% with non-tailored therapy [95% CI 11.6–26.6%]. The median OS for patients treated with a tailored approach was not reached compared to 11.5 months with non-tailored treatment (p=0.016). The 24-month OS for patients treated with a tailored approach was 80.2% [95% CI 40.3–94.8%] compared to 21.1% [95% CI 12.2–31.7%] for patients with non-tailored treatment.

**Conclusions:**

Tailoring of subsequent treatment strategy seems to be essential for achieving long-term benefit. Further studies are required, as the prognosis after CDK4/6i progression remains dismal, especially in cases affecting the central nervous system.

## Introduction

Endocrine treatment in combination with cyclin-dependent kinase 4/6 inhibitors (CDK4/6i) is the standard of care in first-line and second-line treatment of advanced hormone-receptor positive human epidermal growth factor receptor 2 (HER-2) negative advanced breast cancer (1, 2).

Despite the benefits of such therapy, the development of endocrine resistance results in subsequent disease progression (3). Patients who experience a disease recurrence in the first two years of adjuvant endocrine therapy or disease progression in the first six months of endocrine therapy for metastatic breast cancer in the first-line setting are considered to have primary endocrine resistance (1). On the other hand, acquired (secondary) resistance develops later, occurring up to 12 months after completing adjuvant endocrine therapy or more than six months after initiating endocrine therapy in the metastatic setting (1).

The optimal treatment strategy following disease progression on CDK4/6i remains a topic of ongoing debate (4). Understanding endocrine resistance is crucial for optimal management since the response to subsequent therapy may vary significantly depending on its mechanism leading to disease progression (3). The treatment of choice may comprise endocrine therapy, including novel oral selective degraders, such as elacestrant (5) and camizestrant (6), chemotherapy, or new antibody-drug conjugates (7).

Elacestrant has demonstrated improvement in progression-free survival over standard-of-care endocrine monotherapy in CDK4/6i pretreated patients with estrogen receptor 1 (ESR1) gene mutation (5). Nonetheless, despite advancements, more than 40% of patients did not respond to any endocrine therapy in this study.

The addition of alpelisib, the PI3Kα (an alpha isoform of phosphatidylinositol 3-kinase)-specific inhibitor, to fulvestrant has shown progression-free survival (PFS) of 11.0 months in patients with PIK3CA mutated advanced breast cancer who progressed on previous endocrine treatment (8). However, in the phase III SOLAR-1 study, only 5.9% of patients (n=20) with PIK3CA-mutated disease had received CDK4/6i previously (8). In the BYLieve phase 2 trial, comprising patients who progressed on CDK4/6i plus aromatase inhibitor, the median progression-free survival was 7.3 months (9).

A germline mutation in either BRCA1 or BRCA2 gene affects about 5% of people who have breast cancer (10). In that population, poly(adenosine diphosphate-ribose) polymerase (PARP) inhibitors, such as olaparib (11) and talazoparib (12), provided a significant benefit over standard therapy in progression-free survival. Despite no data on the efficacy of PARP inhibitors after progression under CDK4/6i, it is a potential subsequent treatment option.

The choice of systemic therapy mainly depends on the status of estrogen receptor, progesterone receptor, and human epidermal growth factor receptor 2 (HER-2) (2). The discordance of phenotype between primary tumor and metastases and change in phenotype during treatment is an important issue with clinical implications (13). For HER2 status, the negativity of primary and positivity of disease recurrence was observed in almost 14.9% of patients (13). In another study, the rate of change from HER2–0 in primary tumor to HER2-positive in relapse was found to be 1.4%, while from HER2-low to HER2-positive, it was 2.1% (14). Furthermore, HER2 status may change during the treatment of metastatic breast cancer at disease progression; however, more often as a transformation from HER2-positive to HER2-negative disease (15).

Special circumstances need to be considered for proper disease management. Visceral crisis, which is severe organ dysfunction, occurs in around 10%-15% of patients with advanced breast cancer in the first-line setting and requires the most rapidly efficacious therapy (2). Such treatment is also recommended in impending visceral crisis. The term ‘impending visceral crisis’ refers to a precarious state where, although the full criteria for a visceral crisis have not been met, there is a high likelihood of it occurring without prompt and effective intervention (2).

The data regarding optimal treatment after developing resistance to CDK4/6i are inconclusive, and management remains challenging (4). Thus, we aimed to assess predictive and prognostic factors associated with the treatment outcome following CDK4/6i progression.

## Methods

### Study population

We retrospectively analyzed patients who progressed under CDK4/6i treatment between 2018 and 2024 in our cancer center.

We conducted a comprehensive evaluation to determine patients’ survival outcomes following progression on CDK4/6 inhibitors, as well as to identify factors that significantly influence survival rates. The study’s primary endpoint was progression-free survival (PFS) for the treatment following CDK4/6 inhibitor progression. The study’s secondary endpoints were the overall survival (OS) and assessment of the predictive/prognostic value of the duration of response to CDK4/6 inhibitors and progression site.

The Response Evaluation Criteria in Solid Tumours version 1.1 criteria (RECIST v. 1.1) were used to assess the response to treatment. The evaluation classified responses into these categories: complete response (CR), partial response (PR), progressive disease (PD), or stable disease (SD) (16).

Progression-free survival (PFS) was calculated from the beginning of treatment following progression to CDK4/6 inhibitors to the occurrence of disease progression (PD) or death. PFS was calculated only for patients who were eligible for subsequent treatment. Overall survival (OS) was calculated from the beginning of treatment following the CDK4/6 inhibitor to either the patient’s death or the last follow-up. For patients ineligible for subsequent treatment, OS was measured from the date of progression to CDK4/6 inhibitor to either the patient’s death or the last follow-up.

Treatment based on molecular findings (PIK3CA mutation), genetic findings (BRCA1/2 germline mutation), or adapted to the change in the tumor phenotype in rebiopsy (anti-HER2 therapy in the transformation to HER-2-positive disease) was grouped into tailored treatment and compared to the endocrine-based therapy and chemotherapy only.

We acknowledged the definitions of visceral crisis and impending visceral crisis proposed by ABC5 recommendations (2), where a visceral crisis can be described as a critical condition of organ failure, characterized by acute symptoms, diagnostic lab results, and accompanying fast disease progression, and impending visceral crisis.

### Statistical analysis

Categorical variables were presented as frequencies and percentages, while median values were used to show continuous data. The interquartile range (25% to 75%, IQR) measured the data spread. The two groups were compared using either the Fisher exact test or the Wilcoxon rank sum test, according to variable status. Progression-free survival (PFS) and overall survival (OS) were estimated using the Kaplan–Meier method. Survival distributions were compared with a log-rank test. 95% confidence intervals (CIs) were computed for the survival curves. Cox regression was used to assess the effect of proposed factors on PFS and OS. Variables that demonstrated statistical significance in the univariate regression analysis were subsequently included in a multivariate analysis. All tests were two-sided. A p-value ≤ 0.05 showed statistical significance. We performed all tests using Stata Statistical software (version 18, StataCorp, College Station, TX, USA).

## Results

### Study population

Five hundred twelve patients were treated in our center between January 2018 and March 2024. Two hundred six patients were diagnosed with disease progression while on CDK4/6i. Among them, two patients are currently under evaluation for subsequent treatment (PIK3CA assessment), and four patients probably switched to a different cancer center. The remaining 200 patients were included in the analysis. Patients’ characteristics are presented in **Table 1**.

**Table 1:** Patients characteristics.

Characteristics		Patients n=200 (%)
Age	Median, range	64, 24–88
Metastatic disease	*De novo*	73 (37%)
	Recurrent	127 (63%)
Disease Burden before CDK4/6i Tx	Liver Mets	54 (27%)
	Lung/Pleural Mets	80 (40%)
	Bone Mets w/o visc	79 (40%)
CDK4/6i Tx	1^st^ Line	142 (71%)
CDK4/6i compound	Ribociclib	109 (54%)
	Palbociclib	72 (36%)
	Abemaciclib	19 (10%)
Endocrine compound	Aromatase Inhibitor	108 (54%)
	Fulvestrant	92 (46%)
Site of PD	Liver	100 (50%)
	Lung	41 (20%)
	CNS	19 (10%)
	Other*	40 (20%)
Visceral crisis**/**impending visceral crisis		34 (17%)

CDK4/6i, cyclin-dependent kinase 4/6 inhibitors; PD, disease progression according to RECIST 1.1; Mets, metastases; w/o visc – without visceral involvement; CNS, central nervous system; *other includes bones, breast, lymph nodes, peritoneum.

At baseline, 83 patients had a HER2 immunohistochemistry (IHC) score of 0, 69 patients had IHC 1+, and 41 patients had IHC 2+ with negative *in situ* hybridization. Thus, 110 patients represent the population with HER2-low disease. BRCA1/2 status was assessed in 99 patients (49.5%). Twelve patients (6%) had germline BRCA mutation, including four patients with BRCA mutation and eight with BRCA2 mutation. PIK3CA mutational status was tested in 44 patients (22% of patients), and mutation was found in 22 patients (50% of patients tested). Nine patients with PIK3CA mutation were not qualified for alpelisib with fulvestrant treatment, including three patients who received chemotherapy due to visceral crisis and two patients ineligible for any subsequent therapy.

### Progression on cyclin-dependent kinase 4/6 inhibitors

The median overall survival (OS) after progression on CDK4/6i in all patients (n=200) was 9.2 months, with a 12-month OS of 43.7% [95% CI 35.5 – 51.7%].

### Patients ineligible for further treatment

The median age in that group was 69 years (IQR 60–74). The median PFS for CDK4/6i treatment was 15.8 months. At the time of the disease progression, twelve patients had metastases to the liver, fifteen patients to the lung, and six patients experienced disease progression within the central nervous system (CNS). Nine patients had visceral crisis/impending visceral crisis. The significant deterioration in performance status was the main reason for patients’ ineligibility for further treatment. The median OS in patients ineligible for subsequent treatment (n=30) was 2.4 months.

### Patients eligible for further treatment

Among the rest of the patients who received subsequent treatment (n=170), the median OS was 12.7 months, with a 12-month OS of 52.0% [95% CI 42.7 – 60.5%]. Details of subsequent treatments are presented in **Table 2**. Regarding the efficacy of subsequent therapy, the median PFS was 5.4 months. The 6-month PFS was 44.8% (95% CI 36.1 – 53.1%), whereas the 12-month PFS2 was 21.1% (95% CI 14.0 – 29.3%).

**Table 2:** Treatment after progression on CDK4/6 inhibitors.

Treatment	Schedule	Pts [%]
Endocrine-based Tx (non-tailored) N=57 No pts with VC	Fulvestrant	27 [15.9]
	Fulvestrant + Cyclophosphamide (oral, metronomic)	8 [4.7]
	Exemestane + Everolimus	1 [0.6]
	Fulvestrant + Capecitabine (metronomic)	17 [10.0]
	Other (e.g., letrozole, tamoxifen)	4 [2.4]
Chemotherapy only N=94 25 pts with VC	Capecitabine	40 [23.5]
	Paclitaxel	25 [14.7]
	Doxorubicin (Non-pegylated Liposomal) + Cyclophosphamide	16 [9.4]
	Cisplatin/Carboplatin	10 [5.9]
	Other (Pegylated Liposomal Doxorubicin, CMF, Docetaxel)	3 [1.8]
Tailored Tx N=19 No pts with VC	Alpelisib + Fulvestrant	13 [7.6]
	Talazoparib	2 [1.2]
	Trastuzumab + Pertuzumab + Docetaxel	3 [1.8]
	Trastuzumab + Carboplatin	1 [0.6]

Tx, treatment; pts, patients; VC, visceral crisis/impending visceral crisis.

### Central nervous system progression during CDK4/6 inhibitors

**Figure 1:**
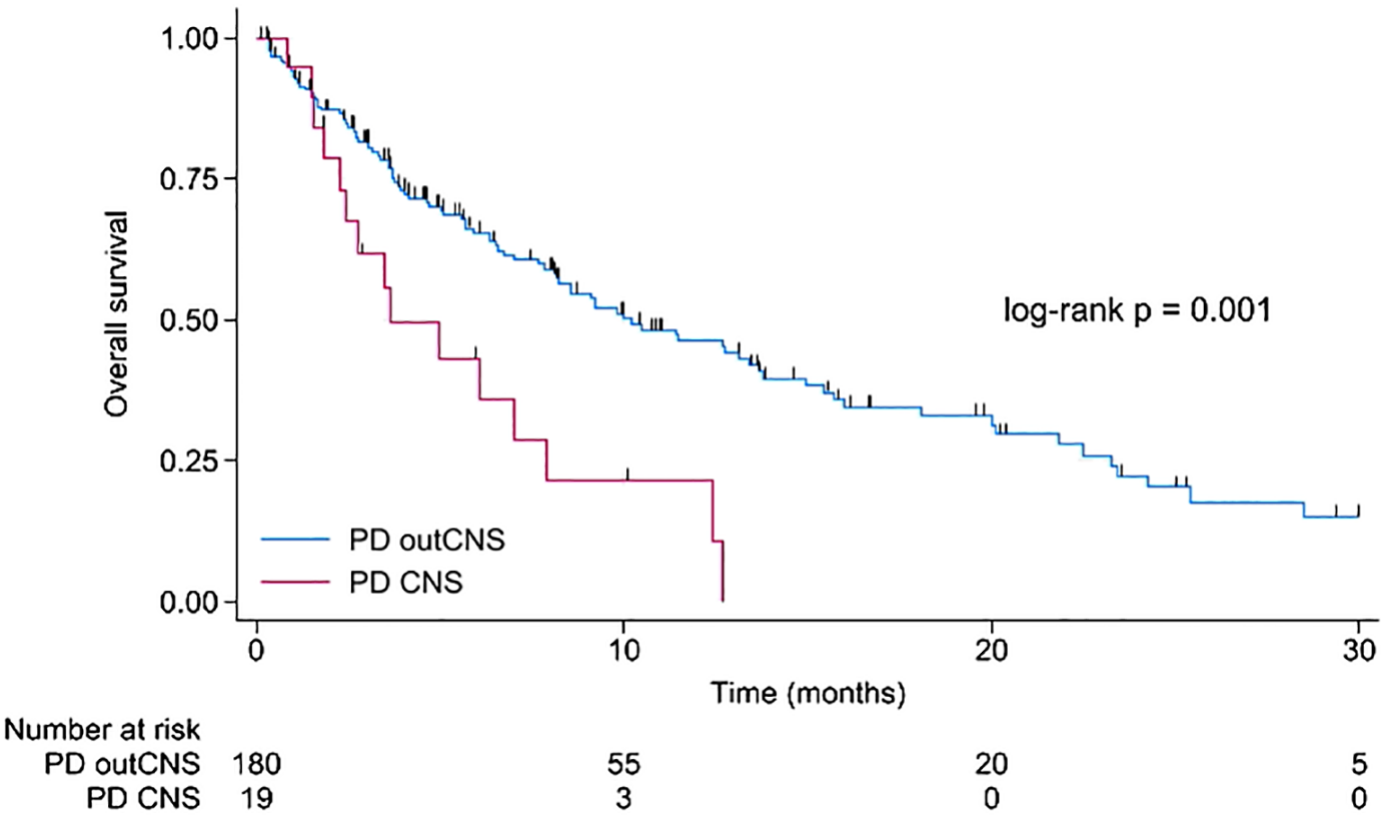
Kaplan-Meier for overall survival for patients with disease progression during CDK4/6 inhibitors in the central nervous system compared to disease progression outside the central nervous system. CNS, central nervous system; PD, disease progression; outCNS, outside the central nervous system.

Nineteen patients had progression in CNS during CDK4/6i treatment, and it was the worst prognostic factor. The median PFS for subsequent treatment in that population was only 2.9 months, while the 6-month PFS was 23.7% (95% CI 3.9 – 52.9%). The median PFS for subsequent treatment in patients with progression outside CNS was 5.6 months, and 6-month PFS was 46.3% (95% CI 37.2 – 54.9%, difference not significant, p = 0.056). Overall survival for patients with CNS progression was significantly shorter than that of patients with progression elsewhere (p=0.001). Results are displayed in **Figure 1**. The median OS was 3.7 months compared to 10.2 months in patients with disease progression outside CNS. The 6-month OS in that population was 43.2% (95% CI 20.1 – 64.5%), compared to 65.5% (95% CI 57.3 – 72.5%) in patients with progression outside CNS. The 12-month OS in patients with PD in CNS was 21.6% (95% CI 5.6 – 44.3%), compared to 46.2% (37.3 – 54.6%) for patients with disease progression outside CNS.

Seven patients received stereotactic radiation therapy. This included six patients who received treatment for the metastatic lesions in CNS, and one patient was irradiated for tumor bed following the surgical resection of a metastatic brain lesion. Nine patients received whole-brain radiotherapy. Three patients were ineligible for local treatment due to a rapid deterioration of performance status. In four patients, CNS progression was accompanied by progression in the liver, two in the lungs, and three in both the lungs and liver. When considering systemic treatment, six patients were deemed ineligible. Two patients received alpelisib in combination with fulvestrant, eight patients chemotherapy (five patients received capecitabine, one paclitaxel, one doxorubicin, and one cisplatin), whereas three patients received endocrine therapy with fulvestrant.

### CDK4/6 inhibitors early progression vs. long response

We compared patients with early progression on CDK4/6i (defined as progression during the first six months of CDK4/6i treatment, n=41) with patients experiencing extended response to CDK4/6i (defined as a response to CDK4/6i for at least 24 months, n=48). Those two groups had no significant differences in response to subsequent treatment (p = 0.416).

### Personalized approach to subsequent treatment

The median PFS in patients treated with endocrine-based therapy (57 patients) was 5.7 months, while in patients treated with chemotherapy only (94 patients), it was 4.3 months (p = 0.440). The median OS in patients treated with endocrine-based therapy was 14.9 months, while in patients treated with chemotherapy only, it was 9.8 months (p = 0.096). Those treatments were grouped as non-tailored therapy.

Treatment based on molecular findings (PIK3CA mutation), genetic findings (BRCA1/2 germline mutation), or adapted to the change in the tumor phenotype in rebiopsy (anti-HER2 therapy in the transformation to HER-2-positive disease) was grouped into tailored treatment. In four patients, transformation to HER2-positive disease in rebiopsy after progression under CDK4/6i was found (from HER2 IHC 0 in 1 patient, from HER2 IHC 1+ in two patients, and from HER2 IHC 2+ and FISH negative in one patient).

**Figure 2:**
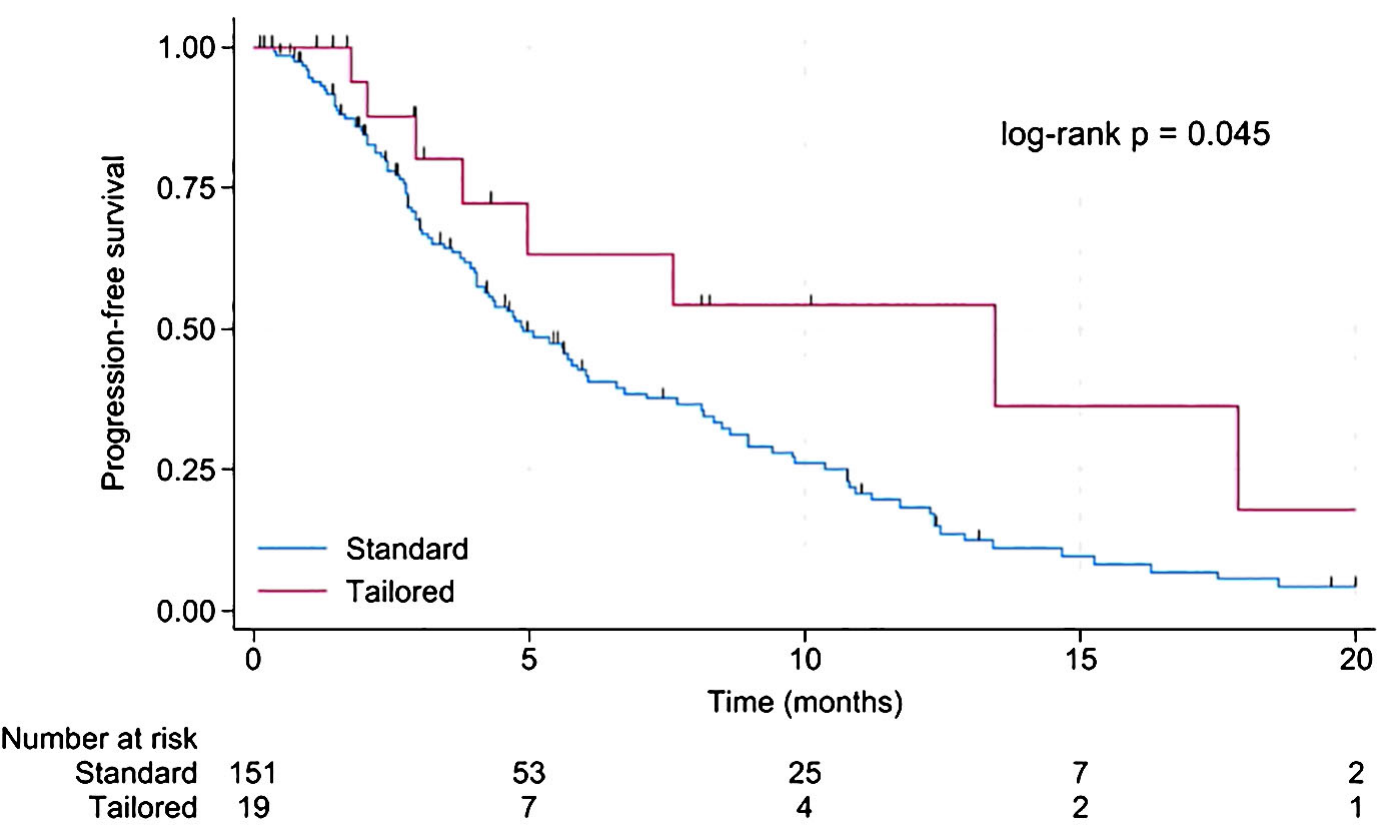
Kaplan-Meier for progression-free survival in patients with standard compared to tailored treatment.

Survival after CDK4/6i progression was significantly longer in patients eligible for tailored treatment. The median PFS in patients with tailored treatment was 13.5 months vs. 4.9 months in patients with non-tailored therapy (p = 0.045). 12-month PFS was 54.1% with tailored treatment [95% CI 24.1 – 76.7%] compared to 18.5% with standard treatment [95% CI 11.6 – 26.6%]. Results are displayed in **Figure 2**. The median follow-up was 6.9 months. In the tailored group, four patients achieved PFS exceeding ten months. Two patients were treated with a combination of docetaxel, trastuzumab, and pertuzumab, while the other two patients were treated with alpelisib plus fulvestrant. Furthermore, a significant majority, accounting for 57.9% (11 patients) within this group, still continued treatment.

As the best response to the treatment, 27 patients achieved partial response, and 62 patients had stable disease. In 70 patients disease progression was found during the first response assessment. Eight patients did not have response evaluation due to a global deterioration of health status requiring treatment discontinuation. Additionally, two patients were before the first response assessment, and one patient was lost to follow-up.

**Figure 3:**
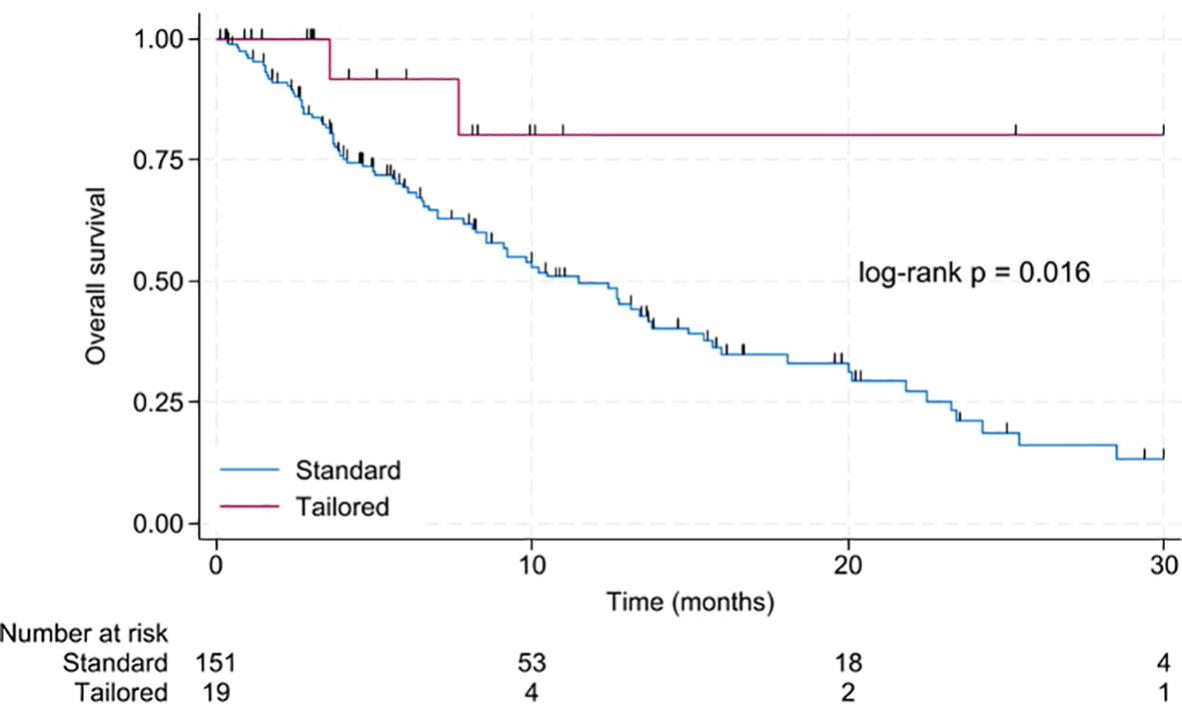
Kaplan-Meier for overall survival in patients with standard compared to tailored treatment.

The median OS for patients treated with a tailored approach was not reached vs. 11.5 months with non-tailored treatment (p = 0.016). The 24-month OS for patients treated with a tailored approach was 80.2% [95% CI 40.3 – 94.8%] compared to 21.1% [95% CI 12.2 – 31.7%] for patients with standard treatment. Results are displayed in **Figure 3**.

## Discussion

The prognosis of patients experiencing disease progression on CDK4/6 inhibitors was unfavorable, particularly regarding the progression in the central nervous system. However, a subset of eligible patients who derived significantly longer survival could be identified when treated with a tailored approach.

Cyclin-dependent kinase 4/6 inhibitors (CDK4/6i) combined with endocrine treatment represent the standard of care for advanced hormone receptor (HR) positive human epidermal growth factor receptor 2 (HER2) negative breast cancer (1). However, there is no consensus on the best treatment option for patients who experienced disease progression while on cyclin-dependent kinase 4/6 inhibitors (CDK4/6i). Randomized clinical trials often comprised only a subset of patients (5, 8). Thus, retrospective analyses that provide real-life data are essential.

In a multicenter, retrospective study, no differences in PFS between endocrine therapy and chemotherapy following progression under CDK4/6i were observed (17). After progression under CDK4/6i in the first line, the median PFS for chemotherapy was 5.3 months and 9.5 months for endocrine treatment, whereas it was 5.7 months and 6.7 months, respectively, after progression under CDK4/6i in the second line (17). Similarly, our study did not find significant differences between these two modalities, with a median PFS of 4.3 months for chemotherapy-only and 5.7 months for endocrine-based treatment. In our study, 25 patients received fulvestrant with metronomic chemotherapy. Such combination have proved some efficacy after CDK4/6i failure (18). Real-world data suggested a survival benefit for continuing a CDK4/6i beyond frontline progression (19). However, in these studies, all patients (20), or at least the vast majority (88.2%) (19), were treated with palbociclib. These findings may not apply to other CDK4/6 inhibitors (21).

The results of rebiopsy after progression under CDK4/6i largely affected treatment decisions in four patients who were diagnosed with transformation to HER2-positive disease and received anti-HER2 therapy. This highlights the fact that rebiopsy should be considered not only when the disease relapses after primary treatment, but also when the disease progresses in the metastatic setting. Nonetheless, proper timing remains crucial, especially in the context of progressive disease. Some biomarker testing to guide treatment selection could be performed during CDK4/6i, such as BRCA1, BRCA2, and PIK3CA mutation assessment, while others rather at disease progression (such as rebiopsy or ESR1 evaluation in ctDNA (5). We recognize, however, that rebiopsy is not suitable for every patient and that not everyone would gain from its findings.

A meta-analysis of eight randomized studies (specifically PALOMA-1, -2, and -3, MONALEESA-2, -3, and -7, and MONARCH-2, and -3) confirmed the results from exploratory analyses that showed that CDK4/6i did not compromise the second progression-free survival (22). Nevertheless, even for patients treated in the pivotal trials, the median PFS for the subsequent treatment was only 6.4 months after the CDK4/6i progression in the first line and 3.8 months after CDK4/6i in the second line (23).

The duration of prior CDK4/6i treatment was found to be positively correlated with response to elacestrant but not to standard-of-care endocrine therapy (24). In our study, we did not observe a significant association between prior CDK4/6i duration and response to subsequent treatment.

Among patients diagnosed with luminal-like breast cancer who developed distant metastases, the frequency of brain metastases is estimated to be approximately 10% (25), similar to our observations. Whether improvements in systemic treatment will result in higher numbers of patients with brain metastases is currently unclear.

The management of brain metastases is still a significant hurdle, even with the advances in systemic treatments for HR-positive HER2-negative metastatic breast cancer (26). We observed that the prognosis of patients who experienced disease progression in the central nervous system under CDK4/6i was dismal. The median overall survival of just 3.7 months highlights a significant unmet clinical need in that population.

Studies regarding the management of patients experiencing disease progression under CDK4/6i often focus only on patients receiving subsequent treatment (17, 19, 20). However, a significant number of patients do not receive any subsequent therapy after CDK4/6i discontinuation. In the MONALEESA-3 trial, among the patients treated with ribociclib in the first-line setting, 36 out of 198 (18.2%) did not receive subsequent treatment. In our study, a comparable proportion of patients were not eligible for further treatment, indicating another subset with an unaddressed medical need.

We acknowledge that our study has several limitations, mostly due to its retrospective nature. Only a small subset of patients in our study received tailored treatment. Furthermore, ESR1 gene mutation has not been assessed in our studied population. Nevertheless, the study provides a rationale for adequate biomarker testing in the population of patients with advanced HR-positive HER2-negative breast cancer and highlights the potential clinical benefits associated with adequate therapy selection.

The median follow-up was just six months. However, given the limited treatment efficacy following CDK4/6i, most patients experienced disease progression within this period.

We acknowledge that baseline factors, including instances of visceral crisis, may bias the observed differences in PFS between the tailored and non-tailored groups. Specifically, after CDK4/6i progression, a visceral crisis or an impending visceral crisis progression was observed in 34 patients, which constitutes 17% of our study population. Within this subset, nine patients were deemed ineligible for further treatment. The remaining 25 patients received chemotherapy. Currently, data on targeted therapy in patients with visceral crisis after CDK4/6i progression are lacking, and such patients were excluded from clinical trials (8, 11, 12).

A BRCA mutation assessment was performed in half of the patients in our study. The percentage of patients with BRCA1/2 germline mutation matched previous studies (10). Two patients were treated with PARP inhibitors after disease progression on CDK4/6i. However, this treatment can be used in later lines. Only 22% had a PIK3CA mutational status assessment, and not all patients with PIK3CA mutation were eligible for targeted treatment. In our study, more than half of our study population had HER2-low status. This is a significant finding since the advent of new anti-HER2 antibody-drug conjugates has broadened the horizon of potential treatments (7).

Trials with novel agents designed to overcome CDK4/6i-resistance are urgently needed (27). However, the population of patients with disease progression under CDK4/6i significantly exceeds the population eligible for most clinical trials (5, 8, 11, 12). Therefore, real-world studies might help improve decision-making for a large number of patients with advanced HR-positive HER2-negative breast cancer experiencing disease progression under CDK4/6i.

The disease’s progression might result from resistance to CDK4/6i (28), endocrine resistance, or a combination of both (3). Mechanisms of resistance to CDK4/6i include loss of Retinoblastoma function (29), upregulation of CDK2 signaling (30), c-MET mutations (31), CDK6 overexpression (32), and activation of PI3K/AKT/mTOR signaling pathway (33). The latter pathway may also contribute to endocrine resistance. Additionally, acquired mutations in ESR1 (34) and NF1 (33) are associated with endocrine resistance.

Despite significant progress, many questions about this complex pathway remain unanswered. Further studies, including patient-derived xenograft (PDX) models (35), evaluation of acquired resistance mechanisms in circulating tumor cells (36), and assessment of genetic alterations in circulating tumor DNA (37, 38), can enhance our understanding and help identify potential novel treatments to overcome resistance.

## Conclusions

Tailoring of subsequent treatment strategy seems to be essential for achieving long-term benefit. Further studies are required, as the prognosis after CDK4/6i progression remains dismal, especially in cases affecting the central nervous system.

## Data availability statement

The original contributions presented in the study are included in the article/**Supplementary Material**. Further inquiries can be directed to the corresponding author.

## Ethics statement

The studies involving humans were approved by Institutional Ethics Committee of Maria Sklodowska-Curie National Research Institute of Oncology, Gliwice Branch; 15 Wybrzeze Armii Krajowej Street, Gliwice, 44 102, Poland. The studies were conducted in accordance with the local legislation and institutional requirements. The ethics committee/institutional review board waived the requirement of written informed consent for participation from the participants or the participants’ legal guardians/next of kin because Retrospective character of the study.

## Author contributions

MK: Conceptualization, Data curation, Formal analysis, Investigation, Methodology, Project administration, Resources, Software, Validation, Visualization, Writing – original draft, Writing – review & editing. AP: Investigation, Writing – review & editing. KŚ: Investigation, Writing – review & editing. AL: Investigation, Writing – review & editing. MM: Investigation, Writing – review & editing. BŁ: Investigation, Writing – review & editing. KC: Investigation, Writing – review & editing. BG: Investigation, Writing – review & editing. NL: Investigation, Writing – review & editing. BB: Investigation, Supervision, Writing – review & editing. EC: Investigation, Writing – review & editing. MJ: Conceptualization, Investigation, Methodology, Resources, Supervision, Validation, Writing – review & editing.

## Supplementary material

SUPPLEMENTARY FIGURE 1 Treatment duration in patients with endocrine-based treatment. Arrows indicate patients who continue treatment.

SUPPLEMENTARY FIGURE 2 Treatment duration in patients treated with the chemotherapy-only approach. Arrows indicate patients who continue treatment.

SUPPLEMENTARY FIGURE 3 Treatment duration in patients with tailored treatment. Arrows indicate patients who continue treatment.
