# Supplementary material for: Tailoring advanced breast cancer treatment after cyclin-dependent kinase 4/6 inhibitors progression - real-world data analysis
Source: Front Oncol. 2024 Jun 7;14:1408664. doi: 10.3389/fonc.2024.1408664 (PMC11190075; doi:10.3389/fonc.2024.1408664)
Supplement: Supplementary file 1 [file Table_1.docx]

**Table 1 Suppl.** Cyclin-dependent kinase 4/6 inhibitors progression-free survival according to subsequent treatment strategy.

| Patients [n=200] | PFS | 24-month PFS | |
| --- | --- | --- | --- |
|  | median | % | 95% CI |
| Ineligible for further treatment [n=30] | 15.8 months | 30.0% | 15.0 – 46.6 |
| Chemotherapy only [n=94] | 10.4 months | 17.0% | 10.3 – 25.3 |
| Endocrine-based treatment [n=57] | 18.1 months | 29.8% | 18.6 – 41.9 |
| Tailored treatment [n=19] | 18.8 months | 31.6% | 12.9 – 52.3 |

**Table 2 Suppl.** Baseline characteristics of patients with tailored treatment compared to patients with a non-tailored approach.

| Characteristics | Tailored Tx [n=19] | Non-tailored Tx [n=151] | p |
| --- | --- | --- | --- |
| Age, median (IQR) | 61 (51-68) | 61 (50-69) | 0.480 |
| Liver metastases | 10 (52.6%) | 88 (58.3%) | 0.633 |
| Lung metastases | 6 (31.6%) | 73 (48.3%) | 0.224 |
| CNS metastases | 3 (15.8%) | 16 (10.6%) | 0.450 |
| VC | 0 | 25 (16.6%) | 0.080 |
| HER2-low | 10 (52.6%) | 86 (58.9%) | 0.628 |
| PIK3CA mutation* | 13 (68.4%) | 7 (4.6%) | <0.001 |
| BRCA 1/2 mutation* | 2 (10.5%) | 9 (6.0%) | 0.354 |

Abbreviations: Tx – treatment; IQR – interquartile range; CNS – central nervous system; VC – visceral crisis/impending visceral crisis

*Patients with the diagnosis of PIK3CA mutation among all cohort.

**Table 3 Suppl.** Univariate Cox regression for progression-free survival.

| Characteristics | HR | 95% CI | p |
| --- | --- | --- | --- |
| Age | 1.01 | 0.99-1.02 | 0.487 |
| De novo metastatic (before CDK4/6i) vs. recurrent | 0.93 | 0.63-1.38 | 0.728 |
| CDK4/6i in the 1^st^ vs. 2^nd^ line | 0.95 | 0.64-1.41 | 0.808 |
| CHT before CDK4/6i vs chemo *naive* | 0.93 | 0.63-1.34 | 0.696 |
| CHT within 1y before CDK4/6i | 0.95 | 0.54-1.67 | 0.868 |
| PD < 6 months on CDK4/6i | 1.26 | 0.1-1.96 | 0.315 |
| PD < 12 months on CDK4/6i | 1.29 | 0.89-1.88 | 0.185 |
| PD < 24 months on CDK4/6i | 1.10 | 0.71-1.70 | 0.677 |
| PD < 36 months on CDK4/6i | 0.86 | 0.43-1.72 | 0.672 |
| PD on CDK4/6i in the liver | 1.09 | 0.97-1.22 | 0.143 |
| PDn on CDK4/6i in the lung | 1.09 | 0.98-1.22 | 0.100 |
| PD on CDK4/6i in the CNS | 2.00 | 0.97-4.15 | 0.062 |
| VC | 1.51 | 0.88-2.57 | 0.132 |

Abbreviations: HR – Hazard Ratio; CHT – chemotherapy; 1y – 1 year; PD – disease progression; CNS – central nervous system; VC – visceral crisis/impending visceral crisis

**Table 4 Suppl.** Univariate Cox regression for overall survival.

| Characteristics | HR | 95% CI | p |
| --- | --- | --- | --- |
| Age | 1.00 | 0.99-1.02 | 0.484 |
| De novo metastatic (before CDK4/6i) vs. recurrent | 0.76 | 0.51-1.13 | 0.171 |
| CDK4/6i in the 1^st^ vs. 2^nd^ line | 1.12 | 0.75-1.65 | 0.586 |
| CHT before CDK4/6i vs chemo *naive* | 1.48 | 1.01-2.19 | **0.046** |
| CHT within 1y before CDK4/6i | 1.13 | 0.62-2.08 | 0.682 |
| PD < 6 months on CDK4/6i | 1.16 | 0.75-1.79 | 0.510 |
| PD < 12 months on CDK4/6i | 1.26 | 0.87-1.83 | 0.229 |
| PD < 24 months on CDK4/6i | 1.13 | 0.70-1.83 | 0.609 |
| PD < 36 months on CDK4/6i | 1.60 | 0.65-3.92 | 0.308 |
| PD on CDK4/6i in the liver | 1.05 | 0.94-1.17 | 0.399 |
| PD on CDK4/6i in the lung | 1.04 | 0.94-1.16 | 0.422 |
| PD on CDK4/6i in the CNS | 2.47 | 1.41-4.30 | **0.002** |
| VC | 2.61 | 1.66-4.09 | **<0.001** |
| Tailored treatment | 0.21 | 0.05-0.86 | **0.030** |

Abbreviations: HR – Hazard Ratio; CHT – chemotherapy; 1y – 1 year; PD – disease progression; CNS – central nervous system; VC – visceral crisis/impending visceral crisis

**Table 5 Suppl.** Cox regression for overall survival.

| Characteristics | Univariate analysis | | | Multivariate analysis | | |
| --- | --- | --- | --- | --- | --- | --- |
|  | HR | 95% CI | p | HR | 95% CI | p |
| CHT before CDK4/6i vs chemo *naive* | 1.48 | 1.01-2.19 | **0.046** | 1.40 | 0.88-2.24 | 0.159 |
| PD on CDK4/6i in the CNS | 2.47 | 1.41-4.30 | **0.002** | 2.34 | 1.10-4.99 | **0.027** |
| VC | 2.61 | 1.66-4.09 | **<0.001** | 1.93 | 1.09-3.40 | **0.024** |
| Tailored treatment | 0.21 | 0.05-0.86 | **0.030** | 0.22 | 0.05-0.89 | **0.034** |

Abbreviations: HR – Hazard Ratio; CHT – chemotherapy; PD – disease progression; CNS – central nervous system; VC – visceral crisis/impending visceral crisis
