# Supplementary figures and images for: Tailoring advanced breast cancer treatment after cyclin-dependent kinase 4/6 inhibitors progression - real-world data analysis
Source: Front Oncol. 2024 Jun 7;14:1408664. doi: 10.3389/fonc.2024.1408664 (PMC11190075; doi:10.3389/fonc.2024.1408664)

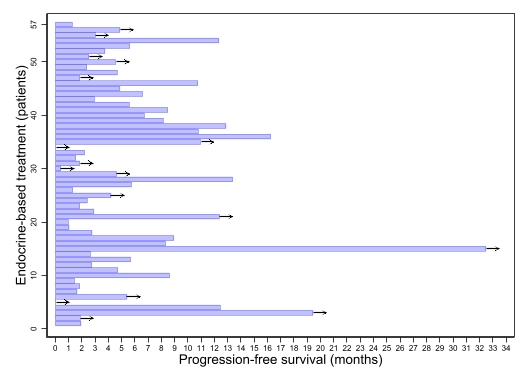

Supplement: SUPPLEMENTARY FIGURE 1 — Treatment duration in patients with endocrine-based treatment. Arrows indicate patients who continue treatment. [file Image_1.jpeg]

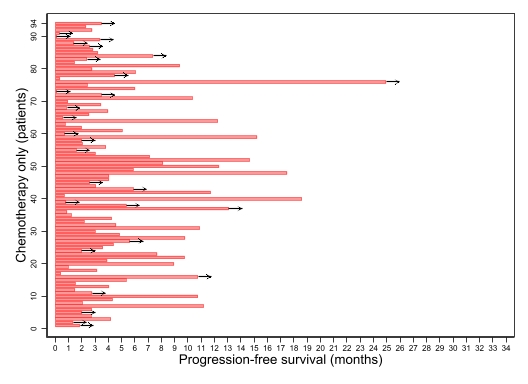

Supplement: SUPPLEMENTARY FIGURE 2 — Treatment duration in patients treated with the chemotherapy-only approach. Arrows indicate patients who continue treatment. [file Image_2.jpeg]

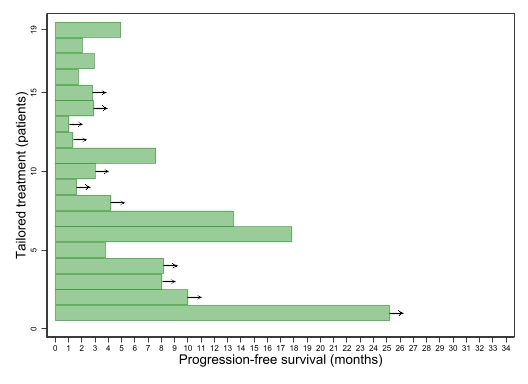

Supplement: SUPPLEMENTARY FIGURE 3 — Treatment duration in patients with tailored treatment. Arrows indicate patients who continue treatment. [file Image_3.jpeg]
